# A retrospective analysis of fibrinolytic and adjunctive antithrombotic treatment during cardiopulmonary resuscitation

**DOI:** 10.1038/s41598-021-03580-6

**Published:** 2021-12-16

**Authors:** Armin Weiss, Christoph Frisch, Rouven Hornung, Michael Baubin, Wolfgang Lederer

**Affiliations:** 1 Department of Anesthesiology and Critical Care Medicine, Medical University of Innsbruck, 6020 Innsbruck, Austria; 2 Department of Anesthesiology and Critical Care Medicine, University Hospital of Innsbruck, 6020 Innsbruck, Austria

**Keywords:** Cardiovascular diseases, Preclinical research, Drug therapy

## Abstract

Synergistic effects of fibrinolytic and additional antithrombotic treatment during cardiopulmonary resuscitation in out-of-hospital cardiac arrest of assumed cardiac origin were evaluated retrospectively. Data were drawn from electronic files of the physician-staffed Emergency Medical Services Tyrol. During a 22-month observation period 53 adult patients were treated with tenecteplase (mean 7641 IU), 19 (32.1%) of whom received additional antithrombotic treatment with heparin (4000–5000 IU) and acetylsalicylic acid (250–500 mg). Lasting return of spontaneous circulation occurred in four of 34 patients who received fibrinolytic treatment only and in seven of 19 patients with additional antithrombotic treatment (*p* = 0.037). Four of five patients who were discharged from hospital had received additional antithrombotic treatment during CPR and were in appropriate neurological status (CPC 1). Considering the small sample size in this retrospective study, the argument may be still be made that fibrinolytic and adjunctive antithrombotic treatment during cardiopulmonary resuscitation in out-of-hospital cardiac arrest of assumed cardiac origin may increase the chances for survival.

## Introduction

Fibrinolytic treatment during cardiopulmonary resuscitation (CPR) is recommended in the current European Resuscitation Council (ERC) Guidelines for Resuscitation in patients suffering out-of-hospital cardiac arrest (OHCA) from suspected or confirmed pulmonary artery embolism (PAE)^1^. However, despite their proven effectiveness many emergency physicians are reluctant to administer fibrinolytics in the out-of-hospital setting^2^. Presumed underlying reasons for the low frequency of fibrinolytic administration are fear of hemorrhage and high costs^3–5^. Another reason might be prolonged CPR of 60 to 90 min that is stipulated by ERC Guidelines when fibrinolytics were administered during resuscitation^1^. Initially, alteplase, a recombinant tissue plasminogen activator (rtPA) and precursor drug of tenecteplase, was conjointly administered with heparin. This adjunctive treatment reduced the frequency of re-thrombosis triggered by split products of fibrin and activated tissue hormones^6^. Contrarily, when tenecteplase was used without adjunctive antithrombotic therapy during advanced life support for OHCA, there was no significant improvement in outcome^7^.

There is evidence that fibrinolytic agents administered during CPR improve cerebral reflow and are associated with increased perfusion and significantly better cerebral function in survivors of OHCA^8,9^. Even in the absence of fibrinolytic treatment, prehospital administration of acetylsalicylic acid (ASA) and heparin was associated with improved survival to hospital discharge and with favorable neurological outcome at hospital discharge^10^.

In this retrospective study we evaluated whether additional administration of heparin and ASA to tenecteplase during CPR was associated with a higher frequency of return of spontaneous circulation (ROSC) and improved outcome.

## Results

**Figure 1:**
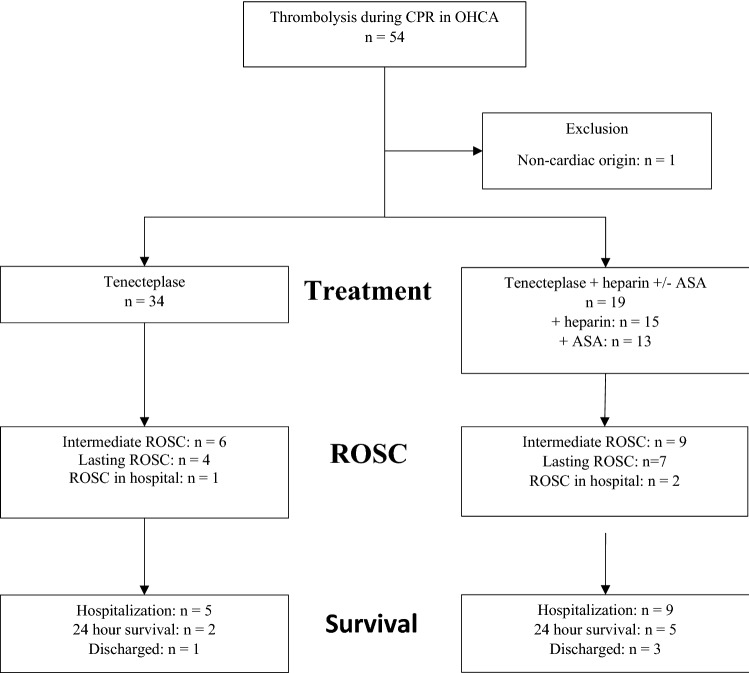
Flow chart showing inclusion of 53 patients with out-of-hospital cardiac arrest of assumed cardiac origin, who were treated with tenecteplase during cardiopulmonary resuscitation.

From July 1, 2016 to April 30, 2018 (22 months) a total of 805 OHCA patients received CPR by ground EMS. During the study period 53 (6.6%) patients were treated with tenecteplase, 19 of whom received additional antithrombotic treatment with ASA and heparin. Median age was 61.0 (range: 31–88) years, 39 (73.6%) were male (Fig. 1).

### Location, bystander and initial cardiac rhythm

Half of emergencies occurred in public places. Bystander CPR was started in 36 (67.9%) patients. Median time to arrival of the emergency physician on site was less than 10 min. Ventricular fibrillation in initial electrocardiography (ECG) recording was observed in approximately one-third of patients (Table 1).

**Table 1:** Characteristics and emergency management of 53 patients with out-of-hospital cardiac arrest of assumed cardiac origin undergoing fibrinolytic treatment, with 19 of them receiving adjunctive antithrombotic treatment.

Characteristics	Tenecteplase	Tenecteplase plus ASA/heparin	*p* value
	(*n* = 34)	(*n* = 19)	
Age, median (IQR), y	60 (34 – 88)	64 (31 – 75)	0.689
Male, *N* (%)	25 (73.5)	14 (73.7)	1.000
*Location*			
Home/private place	15 (44.1)	7 (36.8)	0.991
Public place	16 (47.1)	9 (47.4)	0.957
Other	3 (8.8)	3 (15.8)	0.906
*Bystander*			
Witnessed cardiac arrest, *N* (%)	26 (78.8)	18 (94.7)	0.132
Bystander-initiated CPR, *N* (%)	25 (73.5)	11 (57.9)	0.187
*Initial cardiac rhythm*			
Ventricular fibrillation, *N* (%)	13 (39.4)	7 (36.8)	0.866
Asystole, *N* (%)	9 (27.3)	5 (26.3)	0.951
Pulseless electrical activity, *N* (%)	7 (21.2)	2 (10.5)	0.339
Pulseless ventricular tachycardia, *N* (%)	0	1 (5.2)	0.201
Other, *N* (%)	4 (12.1)	4 (21.1)	0.403
*Complications*			
Hemorrhage *N* (%)	0	2 (20.0)	0.071
Airway management *N* (%)	8 (53.3)	4 (40.0)	0.705
Other *N* (%)	7 (46.7)	4 (40.0)	0.945
*Medication*			
Tenecteplase; mean ± SD, IU	7.067 ± 3.062	8.719 ± 1.032	0.088
Heparin; mean ± SD, IU	0	4.210 ± 2.016	< .001
Acetylsalicylic acid; mean ± SD, mg	0	237 ± 155	< .001
Epinephrine, mean ± SD, mg	9.4 ± 6.6	8.4 ± 7.5	0.350
Amiodarone; mean ± SD, mg	300 ± 112	338 ± 106	0.321
*Ultimate airway management*			
Tracheal intubation, *N* (%)	29 (85.3)	15 (83.3)	0.866
Supraglottic airway, *N* (%)	5 (14.7)	1 (5.6)	0.339
Mask-valve-bag, *N* (%)	0	2 (11.1)	0.053
*Mechanical assist devices*			
LUCAS, *N* (%)	8 (24.2)	4 (21.1)	0.848
AutoPulse, *N* (%)	3 (8.8)	2 (10.5)	0.855
*Return of spontaneous circulation*			
Intermediate, *N* (%)	6 (19.4)	9 (47.4)	0.028
Continuous, *N* (%)	4 (12.9)	7 (36.8)	0.039
None, *N* (%)	21 (67.7)	3 (15.8)	0.001
Transport			
Under CPR, *N* (%)	16 (76.2)	7 (43.8)	0.427
Spontaneous circulation, *N* (%)	5 (23.8)	9 (56.2)	0.013
*Survival*			
24 h, *N* (%)	2 (66.6)	5 (83.3)	0.722
Discharge from hospital, *N* (%)	1 (33.3)	4 (66.6)	0.407

### ROSC and primary survival

Probability of ROSC was significantly higher in patients with additional antithrombotic treatment (Odds Ratio 5.769; *p* = 0.009). Of seven patients with additional antithrombotic treatment who were transported with ongoing CPR, two patients regained spontaneous circulation in the emergency department. Overall, there is a higher probability for primary survival (admission to hospital) in patients who received antithrombotic treatment in addition to tenecteplase (Odds Ratio 3.875, *p* = 0.037). Sixteen (30.2%) of all CPRs were primarily successful (Tab. 1).

### Mechanical assist devices and hospital admission

Mechanical assist devices such as the Lund university cardiac arrest system (LUCAS) and the AutoPulse Resuscitation System were used in 17 patients during rescue (12 cases with LUCAS and five cases with AutoPulse). None of the patients treated with fibrinolytics and a concurrent mechanical assist device survived to discharge. Of seven patients with additional antithrombotic treatment admitted to hospital, two died during the first 24 h. In four patients myocardial infarction was the underlying cause, in one patient necrotizing pancreatitis was diagnosed. Four patients with adjunct antithrombotic treatment (tenecteplase 8.600 IU, heparin 4.500 IU, and ASA 250 mg) survived to discharge, with one of them transferred to another hospital. All survivors had witnessed CA and VF as first rhythm.

### Complications and outcome

Rib fractures were reported in three survivors, sternum fracture in one of them. Liver rupture in one patient necessitated emergency repair under LUCAS resuscitation followed by veno-venous Extracorporeal Membrane Oxygenation (ECMO) for four days. One patient who received heparin during CPR developed HIT II. Four survivors responded four to five years after the incident and were in cerebral performance category (CPC) 1.

## Discussion

In this retrospective study, patients with OHCA of assumed cardiac origin treated with tenecteplase during CPR benefited from additional treatment with ASA and heparin. Lasting ROSC occurred more frequently in patients who received adjunct antithrombotic treatment. To achieve ROSC, coronary perfusion pressure (gradient between diastolic aortic pressure and central venous pressure) must exceed 20 mmHg^11^. We postulate that splitting of fibrinogen correlates with improved coronary perfusion under conditions of low flow tissue perfusion during CPR^12^. Fries et al. observed that microvascular blood flow strongly correlated with coronary perfusion pressure^13^. Presumably, patients who do not respond well to assisted circulation from chest compressions might benefit from fibrinolytic treatment.

Overall, neurologic outcome after pre-hospital cardiac arrest is generally not favorable^14^. Notably, four of five survivors in our study who received antithrombotic treatment in addition to fibrinolytics were in CPC 1, indicating sufficient cerebral function for independent activities of daily life^15^. This is consistent with findings of the matched cohort analysis by Grabmaier et al., who observed that prehospital administration of acetylsalicylic acid and heparin was associated with improved survival to hospital discharge and with favorable neurological outcome at hospital discharge with CPC 1 or 2 classification^8^. We postulate that splitting of fibrinogen correlates with improved microcirculation and fewer regions of no reflow in the brain and thus with better neurologic outcome in survivors^12,16^. Furthermore, heparin and ASA cause sustained prevention of re-thrombosis that is triggered by split products of fibrin and activated tissue hormones^6^.

Amazingly, while the ERC Guidelines for Resuscitation stipulate prehospital thrombolysis in STEMI patients when primary PCI is not possible within 120 min, antithrombotic treatment is not recommended during CPR in OHCA from suspected myocardial infarction^1^. Furthermore, in acute PAE thrombolytic treatment and adjacent administration of unfractionated heparin were shown to significantly reduce the odds of death and recurrence of PAE^2,17^. However, the risk of acute hemorrhage from fibrinolytics, although low, is prevalent. In our study all patients with OHCA and fibrinolytic treatment who underwent chest compressions administered with mechanical assist devices died. Future studies are needed to determine whether concurrent use of fibrinolytic treatment and mechanical assist devices may increase the risk of intrathoracic hemorrhage.

There are several limitations to be considered. Small sample size and the retrospective nature of the study significantly limit the explanatory power of the results. There is a bias of inclusion as we do not know what made the emergency physician on the scene start thrombolytic therapy and administer heparin and ASA. Because of the personal decision by the treating physician on scene, the patients and their individual outcomes are difficult to interpret and to compare. There are numerous confounders, including general health, co-morbidities, pre-existing conditions and medication and confirmed causes of OHCA in the patient population. We do not know the underlying causes of OHCA in the majority of patients, in particular those who remained on scene. Information on underlying causes and adverse effects is restricted to some of the patients who were admitted. Furthermore, the intervals between administration of fibrinolytics, heparin and/or ASA and ROSC were not recorded.

In conclusion, considering the small sample size in this retrospective study, it is still possible to make the argument that fibrinolytic and adjunctive antithrombotic treatment during CPR in OHCA of assumed cardiac origin may increase the chances of survival. There is need of prospective studies to evaluate the effect of fibrinolytic and adjunctive antithrombotic treatment during CPR.

## Materials and methods

A retrospective data analysis of patients with OHCA of assumed cardiac origin treated with tenecteplase during CPR was performed. Effects of additional antithrombotic treatment with ASA and heparin were evaluated.

### Data source, ethical approval and patient consent

All patients with OHCA of assumed cardiac origin in Tyrol (approx. 754.000 population and one of Austria’s nine states) between July 1, 2016 and April 30, 2018, in whom tenecteplase was administered by ground EMS during CPR, were eligible for this retrospective study. Since July 2016 missions performed by Emergency Medical System (EMS) Tyrol have been recorded electronically following the Utstein criteria^18^. Access to data for retrospective analysis was granted by local administration for a study period of 22 months until April 2018. In accordance with the National Code on Clinical Trials the Institutional Ethics Committee (The Ethics Committee of Innsbruck Medical University, Innrain 43, 6020 Innsbruck) waived ethics approval for archived data used in real retrospective studies^19^. The study was conducted according to the guidelines and regulations of the Declaration of Helsinki^20^. Written informed consent was obtained from survivors on the understanding that anonymized information would be published in scientific journals.

### Study setting

In Tyrol, life-threatening emergencies are treated by the physician-staffed EMS. In the case of assumed OHCA the dispatcher calls an ambulance with two emergency assistants close to the location and simultaneously the nearest non-transporting EMS vehicle with one emergency technician and one emergency physician. Whether fibrinolytic treatment is initiated or not is decided by the emergency physician on duty^9^.

Criteria for retrospective patient selection from the database were: adult patients (age ≥ 18 years), OHCA of presumed cardiac origin, administration of tenecteplase with or without additional antithrombotic agents specifically ASA and heparin, treatment by ground EMS during the study period. Exclusion criteria were: incomplete data on fibrinolytic and antithrombotic treatment. Enrolment of cases was following the CONSORT 2010 checklist of trial information^21^.

### Study outcomes

Recently, Grabmaier et al. reported that prehospital administration of ASA and heparin in OHCA was associated with improved survival to hospital discharge and with favorable neurological outcome^8^. Accordingly, we hypothesized that improved outcome after fibrinolytic treatment in cases of OHCA may be associated with even better outcome after fibrinolytic treatment and adjunctive antithrombotic medication. Synergistic effects are anticipated at higher rates of ROSC, admission, hospital discharge and improved cerebral performance category (CPC) in survivors. Primary outcome was ROSC. Secondary outcomes were hospital admission with spontaneous circulation (primary survival) and cerebral performance.

### Statistical analysis

Data analyses were performed using SPSS statistical software, version 26 (IBM Corporation, Armonk, NY, USA). We calculated effect size using relative risk and associated 95% confidence intervals (CI). Odds ratio was applied to measure the association between additional antithrombotic treatment (exposure) and outcome in patients undergoing fibrinolytic treatment during CPR. A probability level of *p* < 0.05 was deemed significant.
